# Prevalence and risk factors of overt postpartum urinary retention among primiparous women after vaginal delivery: a case-control study

**DOI:** 10.1186/s12884-021-04369-1

**Published:** 2022-01-11

**Authors:** Dan Cao, Lin Rao, Jiaqi Yuan, Dandan Zhang, Bangchun Lu

**Affiliations:** Department of Obstetrics and Gynecology, International Peace Maternity and Child Health Hospital affiliated to Shanghai Jiao Tong University School of Medicine, 1961 Huashan Road, Xuhui District, Shanghai, 200030 China

**Keywords:** Postpartum urinary retention, Vaginal delivery, Risk factors, Voiding dysfunction

## Abstract

**Background:**

Postpartum urinary retention (PUR) may lead to bladder neuromuscular damage and subsequently voiding dysfunction. However, the literature regarding the incidence of and risk factors for PUR remains unclear. Moreover, previously reported studies are limited to small sample sizes. Thus, this study aimed to assess the incidence of and risk factors for overt PUR after vaginal delivery.

**Methods:**

This retrospective case-control study included all primiparas who delivered vaginally between July 1, 2017, and June 30, 2019, at our institution. The case group comprised 677 women diagnosed with overt PUR who required catheterisation after delivery. The control group comprised 677 women without overt PUR randomly selected in a 1:1 ratio matched for date of delivery and who delivered immediately after each woman with overt PUR to minimise the impact of variations over time in obstetric practice. Univariate and multivariate logistic regression analyses were performed to investigate the factors associated with overt PUR.

**Results:**

Of the 12,609 women included in our study, 677 were diagnosed with overt PUR (incidence 5.37%). Univariate analysis identified epidural analgesia, episiotomy, perineal tears, instrument-assisted delivery, duration of labour stage, intrauterine operation, and vulvar oedema as risk factors for PUR. Multivariate logistic regression identified epidural analgesia (odds ratio [OR] = 1.41, 95% confidence interval [CI]: 1.11–1.79, *P* = 0.005), vulvar oedema (OR = 6.92, 95% CI: 4.65–10.31, *P* < 0.001), forceps delivery (OR = 8.42, 95% CI: 2.22–31.91, *P* = 0.002), episiotomy (OR = 1.37, 95% CI: 1.02–1.84, *P* = 0.035), and second-degree perineal tear (OR = 3.42, 95% CI: 2.37–4.94, *P* < 0.001) as significant independent risk factors for PUR.

**Conclusions:**

PUR was highly associated with epidural analgesia, forceps delivery, vulvar oedema, episiotomy, and second-degree perineal tears. More attention should be paid to women at high risk to reduce the incidence of PUR.

## Background

Postpartum urinary retention (PUR) is a common postpartum complication characterised by dysuria or a complete inability to urinate after delivery. PUR can result in bladder overdistension, which may lead to bladder neuromuscular damage and subsequently voiding dysfunction [1]. PUR increases the incidence of urinary tract infection and may lead to persistent urinary retention, which substantially affects quality of life [2]. The definition of PUR varies among studies. Yip et al. [3] divided PUR into overt and covert urinary retention, with overt PUR defined as an inability to urinate autonomically 6 h after vaginal delivery or the need for re-catheterisation 6 h after catheter removal following caesarean section. Carley et al. [4] defined PUR as an inability to undergo spontaneous micturition within 12 h after vaginal delivery. However, other reports [5–7] defined PUR as a symptom requiring at least one catheterisation within the first 24 h postpartum. Due to these inconsistencies in the diagnostic criteria, the reported incidence of PUR varies widely from 0.05 to 45% [8–11].

The pathogenesis of PUR is unclear, and research has mainly focused on its high-risk factors. Several studies have reported primiparity, epidural analgesia, instrument-assisted delivery, vaginal or perineal trauma, duration of labour, and neonatal birth weight to be independent risk factors for PUR [12–14]; however, the conclusions are controversial. In our study, we aimed to determine the incidence of overt PUR and identify the risk factors for PUR to provide evidence for the clinical prevention of PUR and reduce the rate of postpartum complications.

## Methods

### Study cohort and data collection

This retrospective matched case-control study included all primiparous women with singletons who had vaginal delivery between July 1, 2017, and June 30, 2019, at the International Peace Maternity and Child Health Hospital. Data were collected through an electronic health record system, in which maternal demographic data, delivery, and postpartum data were recorded in real time. In our study, overt PUR was defined as the need for at least one catheterisation within 12 h postpartum for one or more of the following reasons: 1) patient has not voided within 6 h postpartum and 2) patient experiences incomplete voiding or has an urge to void but cannot void without a residual bladder volume of ≥150 mL as verified by ultrasound scanning or catheterisation within 12 h after vaginal delivery. The inclusion criteria were as follows: 1) primiparous women, 2) single birth, 3) vaginal delivery, and 4) women diagnosed with PUR based on at least one of the above reasons. Women who required an indwelling catheter during labour or within 6 h after delivery for reasons other than urinary retention were excluded from the study. All patients diagnosed with overt PUR were included in the PUR group. The control group comprised primiparas without PUR, who were the first women to deliver immediately after each identified case of PUR, at a 1:1 case-control ratio. Demographic characteristics, delivery information, and postpartum complications of the abovementioned cases were collected and analysed. Vulvar oedema was considered when dermatoglyphs had disappeared, and the swollen skin was 1 cm higher than that of healthy perineal skin and more than 2 cm in length, as examined by a trained nurse.

According to the literature, the incidence of overt PUR is 0.14–9.85% (approximately 5% on average), whereas the incidence of overt PUR in normal women is 0.07 per 1000 women [15]. We used PASS (Power Analysis and Sample Size) (v 15.0.5) to estimate the sample size. Based on a target power of 90% and an α error of 5%, the target size was calculated to be 201 women. Our study included 677 pairs of patients and normal controls, which was substantially more than the calculated minimal target size. Thus, we believe that the sample size of the current study is adequate to investigate the condition of overt PUR.

The hospital protocol on detecting and managing postpartum urinary retention was performed. If the puerpera did not void within 4 h after vaginal delivery, non-invasive measures were commenced as follows: listening to the sound of running water, warm shower, and abdominal hot compress physiotherapy. We waited for further 2 h after performing the non-invasive measures. If by 6 h after delivery, the puerpera was still unable to urinate or the postvoid residual bladder volume (PVRBV) was ≥150 mL, overt PUR was diagnosed and an indwelling catheter (IDC) was inserted for 12 h. Six hours after removal of the IDC, if inability to void was noted or urination was unsatisfactory (PVRBV ≥150 mL), an IDC was inserted for additional 48 h. The resolution of PUR was defined as satisfactory urination or a PVRBV of < 150 mL. A PVRBV of > 150 mL was identified by catheterisation or by transabdominal ultrasound screening. Ultrasound scanning was performed by a doctor experienced in ultrasound examination using a Colour Doppler Ultrasonic Diagnosis Apparatus (Voluson E6, GE, USA). The PVRBV was calculated using the following formula for approximation of the ellipsoid: volume = π (D1 × D2 × D3) / 6, where D1 is the widest diameter in transverse plane in cm, D2 is the anteroposterior diameter in the longitudinal plane in cm, and D3 is the cephalocaudal diameter in longitudinal plane in cm, and π = 22/7).

### Statistical analysis

Continuous variables are presented as means (standard deviation) and were analysed using a two-sample t-test. Categorical variables are presented as percentages, and were compared using the Chi-square (χ^2^) test. Multiple logistic regression was performed to identify factors independently associated with a high risk of developing PUR. A forward stepwise variable selection procedure was used. Odds ratios (ORs) and 95% confidence intervals (CIs) for independent risk factors were calculated. Missing data points were excluded from the analysis. SPSS v23.0 (IBM Corp., Armonk, NY, USA) was used for the statistical analysis. Statistical significance was set at a *P*-value of < 0.05.

## Results

A total of 12,609 primiparous women who underwent vaginal deliveries were included. Of these 12,609 women, 677 experienced overt PUR (incidence 5.37%). Among these 677 women with overt PUR, 573 (84.6%) and 55 (8.1%) recovered urination function after catheterisation within 48 and 72 h, respectively. A total of 41 patients (6.1%) required catheterisation for > 72 h but recovered within 7 days. Eight patients were diagnosed with persistent PUR and required catheterisation for > 7 days; they were discharged with catheters and returned for catheter removal 7 days later. Eventually, PUR was resolved in all patients.

Table 1 describes the baseline and obstetric characteristics of the cohort. No significant differences were found in age, gestational age, pre-gestational body mass index, or weight gain during pregnancy between the two groups. Univariate analysis revealed significant differences in the proportions of epidural analgesia (63.8% vs. 52.9%, *P* < 0.001), episiotomy (44.9% vs. 25.1%, *P* < 0.001), second-degree perineal tear (43.7% vs. 42.8%, *P* < 0.001), forceps delivery (19.9% vs. 5.9%; *P* < 0.001), and vulvar oedema (27.9% vs. 4.9%; *P* < 0.001) in the PUR and control groups. Parturients with a longer first or second stage of labour were more likely to experience PUR than those with a shorter first or second stage of labour (51.2% vs. 43.6%, *P* = 0.001 and 41.6% vs. 30%, *P* < 0.001, respectively).

**Table 1:** Baseline and obstetric characteristics of the two groups

Variable	PUR group (*n* = 677)	control group (*n* = 677)	*P* value
Age (years)	29.55 (3.12)	29.23 (3.15)	0.064
Pre-gestational BMI (kg/m^2^)	20.74 (2.50)	20.62 (2.58)	0.402
Weight gain (kg)	14.35 (4.30)	14.31 (4.39)	0.849
Gravidity			0.238
One	501 (74)	482 (71.2)	
Two	147 (21.7)	153 (22.6)	
Three or more	29 (4.3)	42 (6.2)	
Gestational age at delivery (weeks)	39.15 (1.32)	39.14 (1.28)	0.890
Neonatal birth weight (g)			0.113
< 3000	136 (20.1)	156 (23.0)	
3000–3499	325 (48.0)	338 (49.9)	
≥ 3500	216 (31.9)	183 (27.0)	
Epidural analgesia	432 (63.8)	358 (52.9)	< 0.001
Intact perineum	7 (1.0)	20 (3.0)	< 0.001
1st degree	70 (10.3)	197 (29.1)	
2nd degree	296 (43.7)	290 (42.8)	
Episiotomy	304 (44.9)	170 (25.1)	
Forceps delivery	138 (19.9)	40 (5.9)	< 0.001
Duration first stage (min)			0.001
< 360	251 (37.1)	319 (47.1)	
360–719	347 (51.2)	295 (43.6)	
≥ 720	79 (11.7)	63 (9.3)	
Duration second stage (min)			< 0.001
< 60	381 (56.3)	461 (68.1)	
60–179	282 (41.6)	210 (30.0)	
≥ 180	14 (2.1)	6 (0.9)	
Intrauterine operation	64 (9.4)	83 (12.3)	0.097
Vulvar edema	189 (27.9)	33 (4.9)	< 0.001

Data are presented as mean (SD) or n (%); *BMI* body mass index

Multiple logistic regression analysis showed that epidural analgesia (OR = 1.41, 95% CI: 1.11–1.79, *P* = 0.005), vulvar oedema (OR = 6.92, 95% CI: 4.65–10.31, *P* < 0.001), forceps delivery (OR = 8.42, 95% CI: 2.22–31.91, *P* = 0.002), episiotomy (OR = 1.37, 95% CI: 1.02–1.84, *P* = 0.035), and second-degree perineal tear (OR = 3.42, 95% CI: 2.37–4.94, *P* < 0.001) were significant independent risk factors for PUR. However, the length of the first and second stages of labour had no independent effect on the risk of PUR (Table 2).

**Table 2:** Multiple logistic regression model for risk factors of PUR

Variable	Odds ratio	95% CI	*P* value
Epidural analgesia	1.41	1.11–1.79	0.005
Vulvar edema	6.92	4.65–10.31	< 0.001
Forceps delivery	8.42	2.22–31.91	0.002
Episiotomy	1.37	1.02–1.84	0.035
Second-degree perineal tear	3.42	2.37–4.94	< 0.001

## Discussion

According to recent reports, PUR is classified as overt, covert, and persistent PUR [1, 3, 16, 17]. Overt PUR is symptomatic, requires treatment, and may result in persistent PUR in case of inadequate management, while covert PUR is asymptomatic and mostly self-healing. Therefore, our study focused on overt PUR. In the past 20 years, the incidence of overt PUR has been reported to be as low as 0.14% [18], while Gema et al. reported a higher incidence of 9.85% [14]. This large variation in morbidity may be due to the different definitions of overt PUR and inclusion criteria, such as including women who deliver via caesarean section [19]. Compared with primiparas, multiparas who have a shorter labour process, and lower rates of analgesic delivery, and proportions of instrument-assisted delivery may have a higher incidence of PUR [20]. We avoided the confounding effect of multiparas by restricting the study population to primiparous women. Our results indicated that the incidence of overt PUR was 5.37%, which was similar to the 4.9% incidence reported by Yip et al. [3]. Furthermore, eight patients eventually developed persistent PUR (incidence 0.06%), which is in concordance with the incidence of 0.05% reported by Zussman et al. [21]. The results showed that most patients with PUR experience recovered bladder function within 7 days and that persistent PUR is a rare postpartum complication [22].

The specific aetiology of PUR is not well understood. Multiple factors related to pregnancy and labour may cause muscle and nervous tissue damage, which can ultimately lead to PUR [1, 3]. In previous studies, univariate analyses confirmed that PUR is commonly associated with epidural analgesia, prolonged stages of labour, episiotomy, instrumental delivery, perineal tears, vulvar oedema, neonatal birth weight, and other factors [4, 6, 7, 9]. However, the results are inconsistent regarding independent risk factors [6, 13, 14, 18, 19]. Our findings revealed that epidural analgesia, vulvar oedema, forceps delivery, episiotomy, and second-degree perineal tears were independent factors. These differences may be related to variations in study sample sizes, study designs (such as excluding the multiparous women), populations, or practice protocols.

Consistent with previous reports [4, 12, 14], epidural analgesia was considered an independent risk factor for PUR in our study. This is because epidural analgesia directly affects bladder sensitivity and contractility [3, 23]. Foon et al. [24] suggested that motor and sensory functions are affected differently by analgesia. Epidural anaesthesia can affect afferent inputs and suppress sensory stimuli from the bladder to the pelvic and lower abdominal nerves. Subsequently, detrusor muscle contraction and urethral relaxation become out of sync, which affects normal micturition [4, 9, 25]. This results in overdistension of the bladder and eventually urinary retention. Simmons et al. [26] suggested that the combined use of opioids and lumbar and epidural anaesthesia increases the risk of PUR. This indicates that opioids applied during epidural anaesthesia may have a decisive influence on PUR.

Our study confirmed the role of forceps delivery as a risk factor for PUR, which was previously reported [4, 12, 20, 25, 27]. Instrument-assisted delivery may damage the pelvic, pudendal, and peripheral nerves, resulting in impairment of the reflexes and voluntary mechanisms required for urination [4, 25], as well as mechanical outlet obstruction due to vulvar oedema or hematoma. Direct bladder or urethral trauma resulting from instrument-assisted delivery contributes to PUR [28]. Our study indicated that vulvar oedema increased the risk of PUR by up to 6.92-folds, which corroborates this statement. In addition, pain caused by vulvar oedema may result in reflex urethral spasm, and subsequently PUR [4, 29].

In the present study, episiotomy and second-degree perineal tears were both identified as independent risk factors for PUR, which is in line with the findings of previous studies [6, 19, 27, 30, 31]. In a systematic review by Mulder et al. [12], episiotomy was found to be directly related to PUR. Avondstondt et al. [32] reported that second-degree or worse obstetrical laceration increased the risk of PUR by 3.66-folds. The mechanism is yet to be determined, but it may be that pain caused by perineal trauma leads to changes in bladder sensation, central nervous system inhibition, and persistent urethral spasm [33]. However, there is a lack of consensus in the literature regarding the independent contribution of episiotomy to PUR [6, 20, 25]. Therefore, it is still questionable whether episiotomy is an independent risk factor. Buchanan et al. [13] reported that only third- or fourth-degree perineal trauma was a significant independent predictor of PUR. There were no cases of third- or fourth-degree perineal trauma in our study. Severe perineal trauma is a rare condition, and even in our hospital with a large delivery number, there are less than five cases per year. In addition, patients with severe perineal trauma typically require catheterisations after suturing, which was not one of our inclusion criteria.

One of the strengths of this study is the inclusion of a large homogeneous population of primiparous women. We excluded multiparous women who experienced a fast labour process, lower rate of perineal trauma, and instrument-assisted delivery to avoid confounding effects. Because our hospital is one of the largest obstetrics and gynaecology hospitals in Shanghai, a populous super city, with > 15,000 deliveries annually, to the best of our knowledge, this study has the largest sample size as a study of primiparous women with PUR; therefore, it can represent the whole population to a certain extent.

However, this study has some limitations. Primarily, it was performed at a single centre and was designed as a retrospective study, which made it impossible to assess causality. Second, other factors, such as catheterisation during labour, may have contributed to the risk factors. Neron et al. [34] reported that intermittent bladder catheterisation immediately postpartum could reduce the risk of PUR. Another study [32] found that intermittent catheterisation during labour was independently associated with an increased risk of PUR. In our hospital, the rate of catheterisations during labour was not well documented; therefore, it was not included in our study. In the future, a prospective study is necessary to clarify the risk factors for PUR.

## Conclusions

PUR was highly associated with epidural analgesia, forceps delivery, vulvar oedema, episiotomy, and second-degree perineal tears. More attention should be paid to women at high risk to reduce the incidence of PUR.

## Data Availability

The datasets used and/or analysed during the current study are available from the corresponding author on reasonable request.

## Abbreviations

BMI: Body mass index
CI: Confidence interval
cm: Centimetre
IDC: Indwelling catheter
mL: Millilitre
OR: Odds ratio
PUR: Postpartum urinary retention
PVRBV: Postvoid residual bladder volume
